# Associations of early pregnancy serum uric acid levels with risk of gestational diabetes and birth outcomes: a retrospective cohort study

**DOI:** 10.1186/s12902-023-01502-3

**Published:** 2023-11-20

**Authors:** Ting-Ting Pang, Zi-Xing Zhou, Peng-Sheng Li, Hui-Ting Ma, Xiu-Yin Shen, Ying-Chun Wan, Xiao-Ling Guo, Zheng-Ping Liu, Geng-Dong Chen

**Affiliations:** 1 Department of Medical Records, Foshan Women and Children Hospital, Foshan city, Guangdong Province 528000 China; 2 Department of Obstetrics, Foshan Institute of Fetal Medicine, Foshan Women and Children Hospital, Foshan city, Guangdong Province 528000 People’s Republic of China

**Keywords:** Uric acid, Early pregnancy, Gestational Diabetes Mellitus, Preterm birth, Birth weight

## Abstract

**Background:**

Previous evidence suggests that higher blood uric acid (UA) levels are associated with adverse cardiovascular outcomes during pregnancy and subsequent birth outcomes. However, it has been relatively unclear whether these associations persist in normotensive pregnant women.

**Methods:**

The study was based on a retrospective analysis of 18,250 mother-infant pairs in a large obstetric center in China. Serum UA concentrations in early pregnancy (median: 17.6, IQR: 16.3, 18.6 gestational weeks) were assessed. Hyperuricemia was defined as ≥ one standard deviation (SD) of the reference value for the corresponding gestational age. Outcomes of gestational diabetes mellitus (GDM), preterm birth (PB), low birth weight (LBW), macrosomia, small for gestational age (SGA) and large for gestational age (LGA) were extracted from the medical records.

**Results:**

The mean maternal UA level was 0.22 ± 0.05 mmol/L, and 2,896 (15.9%) subjects had hyperuricemia. After adjustment for several covariates, UA was associated with several adverse outcomes. The ORs (95%CI) per one SD increase in serum UA concentration were 1.250 (1.136, 1.277) for GDM, 1.137 (1.060, 1.221) for PB, 1.134 (1.051, 1.223) for LBW, and 1.077 (1.020, 1.137) for SGA, respectively. Similar adverse associations were found between hyperuricemia and GDM, PB (ORs: 1.394 and 1.385, P < 0.001), but not for LBW, macrosomia, SGA, and LGA. Adverse associations tended to be more pronounced in subjects with higher BMI for outcomes including PB, LBW, and SGA (P interaction = 0.001–0.028).

**Conclusion:**

Higher UA levels in early pregnancy were associated with higher risk of GDM, PB, LBW, and SGA in normotensive Chinese women.

**Supplementary Information:**

The online version contains supplementary material available at 10.1186/s12902-023-01502-3.

## Background

Uric acid (UA) is one of the major factors that were associated with an increased risk of cardiovascular disease [1, 2]. Evidence suggests that higher UA levels also contribute to other chronic diseases such as type 2 diabetes mellitus [3], metabolic syndrome [4], and non-alcoholic fatty liver disease [5]. During pregnancy, higher UA levels are known to be associated with an increased risk of gestational hypertension, pre-eclampsia/eclampsia [6–8], and subsequent adverse birth outcomes [9]. However, the associations between UA and other complications, such as gestational diabetes mellitus (GDM) and adverse birth outcomes, have been relatively unclear in normotensive subjects. In addition, previous studies in this area have produced inconsistent results. Several studies have suggested that hyperuricemia is associated with an increased risk of GDM [10–13] or adverse birth outcomes [14–16]. One study showed that both high and low UA levels contribute to adverse fetal growth [17]. Marginal or null associations with these outcomes have been reported for UA in several other studies [18–22]. The small sample size of most studies, racial heterogeneity, and different trimesters studied may partially explain the heterogeneity of these findings. However, it should be recognized that gestational cardiovascular complications (hypertension, pre-eclampsia, eclampsia) share many common mechanisms with GDM, and independently lead to adverse birth outcomes. Therefore, without eliminating the influence of these patients, the associations of UA with other outcomes in the general population may be overestimated. In addition, with the potential function of antioxidant, UA has also been suggested to be beneficial for degenerative diseases such as osteoporosis [23], and showed U-shaped associations for several diseases [24–26]. A U-shaped rather than linear association of UA with fetal growth has been reported [17]. The functional forms of UA with pregnancy outcomes have been inconsistent or poorly investigated in other studies and warrant further investigation.

Therefore, we aimed to perform a retrospective analysis based on medical record data from a large obstetric center in southern China to better illustrate these problems.

## Methods

### Subjects

**Fig. 1:**
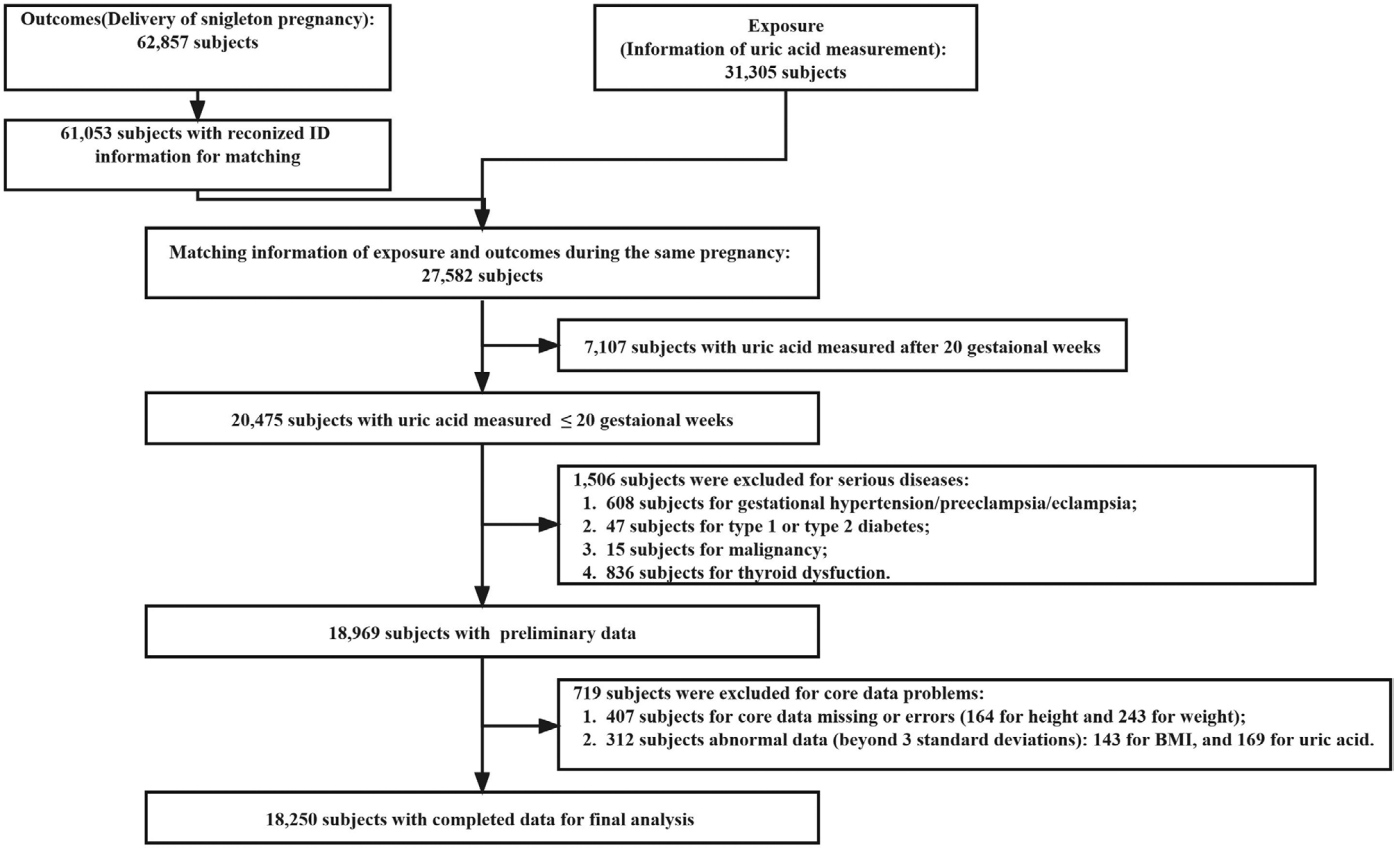
Flow characteristic of the study

The study was based on retrospective data from the Southern Medical University Affiliated Maternal & Child Health Hospital of Foshan from January 1, 2012 to July 31, 2018. The hospital is the largest obstetric center in Foshan City, Guangdong Province, China, covering a large population of more than 7.7 million people. Singleton pregnant women were included in the analyses if they had serum UA measured at antenatal care visits during the first 20 weeks of pregnancy and delivered a live birth at the hospital. A total of 27,582 subjects provided matching information on exposures and outcomes during the same pregnancy. Subjects were further excluded if they met the following criteria: (a) with UA concentrations detected after 20 weeks’ gestation (7,107 subjects); (b) a history of serious medical conditions: including gestational hypertension/pre-eclampsia/eclampsia (608 subjects), type 1 or type 2 diabetes (47 subjects), malignancy (15 subjects), and thyroid dysfunction (836 subjects); (c) missing core data (407 subjects) or suspicious outliers (> 3SD or <-3 SD, 312 subjects). Finally, a total of 18,250 mother-infant pairs were included in the analyses (Fig. 1).

### Data collection

Serum UA concentration data were obtained from clinical laboratory records or medical records. Blood samples were collected during routine obstetric examinations and measured immediately by the clinical laboratory at the hospital without freezing. UA was detected by the uric acid enzyme colorimetric method using an automated biochemical analyzer (AU5800, Beckman Coulter, Inc, USA). Commercial kits were purchased from Ningbo Ruiyuan Biotechnology Co., Ltd, China. The intra- and inter-assay coefficients of variation were less than 4% and 15%, respectively. To reduce the possibility of influence from pregnancy complications, we restricted the data to UA measured before the first 20 weeks of gestation. Hyperuricemia in early pregnancy is defined as serum UA concentrations ≥ 1 SD of reference values [27] at the gestational ages (6 ~ 12, > 12 ~ 13, > 13 ~ 14, > 14 ~ 15, > 15 ~ 16, > 16 ~ 17, > 17 ~ 18, > 18 ~ 19, and > 19 ~ 20 gestational weeks) in our study, as shown in Supplemental Table 1.

Information including maternal age, body mass index (BMI), gestational week of UA measurement, parity, time of last menstrual period and delivery, history of related diseases were obtained from medical records. The outcomes of GDM, PB, LBW, and macrosomia were diagnosed by professional obstetricians with the same criteria, and extracted from medical records. GDM was assessed by the oral glucose tolerance test at 24 to 28 weeks’ gestation, and diagnosed if subjects met any items of the following criteria: fasting blood glucose ≥ 5.1 mmol/L; one-hour blood glucose after oral glucose ingestion ≥ 10.0 mmol/L; two-hour blood glucose after oral glucose ingestion ≥ 8.5 mmol/L. PB was defined as delivery at ≥ 28 and < 37 weeks; gestation. Neonatal birth weight < 2500 g was defined as LBW, and ≥ 4000 g was defined as macrosomia. Based on the latest criteria (WS/T 800—2022) promulgate by the National Health Commission of the People’s Republic of China [28], appropriate for gestational age (AGA) was defined as neonates whose birth weights were between 10th to 90th percentile by gestational age. Small for gestational age (SGA) and (LGA) was defined as neonates whose birth weights were < 10th percentile and > 90th percentile by gestational age, respectively.

### Statistical analyses

Continuous variables were presented as mean ± standard deviation (SD) or median (interquartile range) and tested by Student’s t-test or non-parametric test. Categorical variables were presented as frequencies (percentages) and tested by chi-squared test. Logistic regression analyses were performed to examine the associations between UA and outcomes, including GDM, PB, LBW, macrosomia, SGA, and LGA. UA was analyzed as both a categorical (hyperuricemia versus normal) and continuous variable (per one SD increase). Two different analysis models were performed, model 1 as a univariate model without any adjustment and model 2 adjusted for maternal age, BMI, parity, and gestational week of UA measurement. Stratified analyses were performed for different groups of maternal age (< 35 or ≥ 35 years), BMI (< 24, overweight: ≥24 and < 28, obesity: ≥28 kg/m^2^) [29], parity (0 or ≥ 1), gestational week of UA measurement (< 17 or ≥ 17 weeks). Analyses were performed with SPSS software (version 21.0, Chicago, IL, USA). Generalized additive regression models (GAMs) were used to explore the functional forms of the association between UA and related outcomes and were performed using the R software (version 4.3.1, Vienna, Australia). A two-sided P < 0.05 was considered as statistically significant.

## Results

A total of 18,250 pregnant women aged 29.2 ± 4.53 years were included in the analyses, of whom 2,896 (15.9%) had hyperuricemia. As shown in Table 1, maternal serum UA concentrations were measured at a median of 17.6 (IQR: 16.3, 18.6) weeks, with a mean value of 0.22 ± 0.05 mmol/L. The incidence was highest for LGA (13.2%), followed by SGA (9.2%), GDM (7.6%), PB (5.0%), LBW (4.4%), and lowest for macrosomia (2.4%). Subjects with hyperuricemia tended to have higher BMI (26.6 vs. 25.8 kg/m^2^, P < 0.001) and serum UA concentration (0.30 vs. 0.21 mmol/L, P < 0.001); higher incidence of LGA (15.0% vs. 12.9%, P = 0.007), GDM (9.8% vs. 7.2%, P < 0.001), CS (45.8% vs. 42.7%, P = 0.002), and preterm birth (6.1% vs. 4.8%, P = 0.004); and have a lower delivery gestational age (38.7 vs. 38.9 weeks’ gestation, P < 0.001).

**Table 1:** Characteristic of subjects

	Total (N = 18,250)	Normal (N = 15,354)	Hyperuricemia (N = 2,896)	*P*
Age, years	29.2 ± 4.53	29.2 ± 4.52	29.2 ± 4.60	0.717
BMI,kg/m^2^	26.0 ± 2.96	25.8 ± 2.90	26.6 ± 3.20	**< 0.001**
Gestational age at delivery, weeks	39.2 (38.2, 40.1)	39.2 (38.3, 40.1)	39.1 (38.2, 39.9)	**< 0.001**
Neonatal birth weight, kg	3.17 ± 0.42	3.17 ± 0.42	3.16 ± 0.43	0.085
Uric acid, mmol/L	0.22 ± 0.05	0.21 ± 0.04	0.31 ± 0.03	**< 0.001**
Gestational weeks of uric acid measurement, weeks	17.6 (16.3, 18.6)	17.6 (16.3, 18.6)	17.6 (16.3, 18.6)	0.520
Parity, N (%)				0.06
0	12,932 (70.9)	10,922 (71.1)	2,010 (69.4)	
≥1	5,318 (29.1)	4,432 (28.9)	886 (30.6)	
Gestational diabetes mellitus, N (%)				**< 0.001**
Yes	1,396 (7.6)	1,113 (7.2)	283 (9.8)	
No	16,854 (92.4)	14,241 (92.8)	2,613 (90.2)	
Caesarean section, N (%)				**0.002**
Yes	7,890 (43.2)	6,563 (42.7)	1,327 (45.8)	
No	10,360 (56.8)	8,791 (57.3)	1,569 (54.2)	
Premature birth, N (%)				**0.004**
Yes	918 (5.0)	741 (4.8)	177 (6.1)	
No	17,332 (95.0)	14,613 (95.2)	2,719 (93.9)	
Low birth weight, N (%)				0.546
Yes	812 (4.4)	677 (4.4)	135 (4.7)	
No	17,438 (95.6)	14,677 (95.6)	2,761 (95.3)	
Macrosomia, N (%)				0.722
Yes	443 (2.4)	370 (2.4)	73 (2.5)	
No	17,807 (97.6)	14,984 (97.6)	2823 (97.5)	
Weight for gestational age				**0.007**
Small for gestational age, SGA	1,671 (9.2)	1,401 (9.1)	270 (9.3)	
Appropriate for gestational age, AGA	14,171 (77.6)	11,978 (78.0)	2,193 (75.7)	
Large for gestational age, LGA	2,408 (13.2)	1,975 (12.9)	433 (15.0)	

**Fig. 2:**
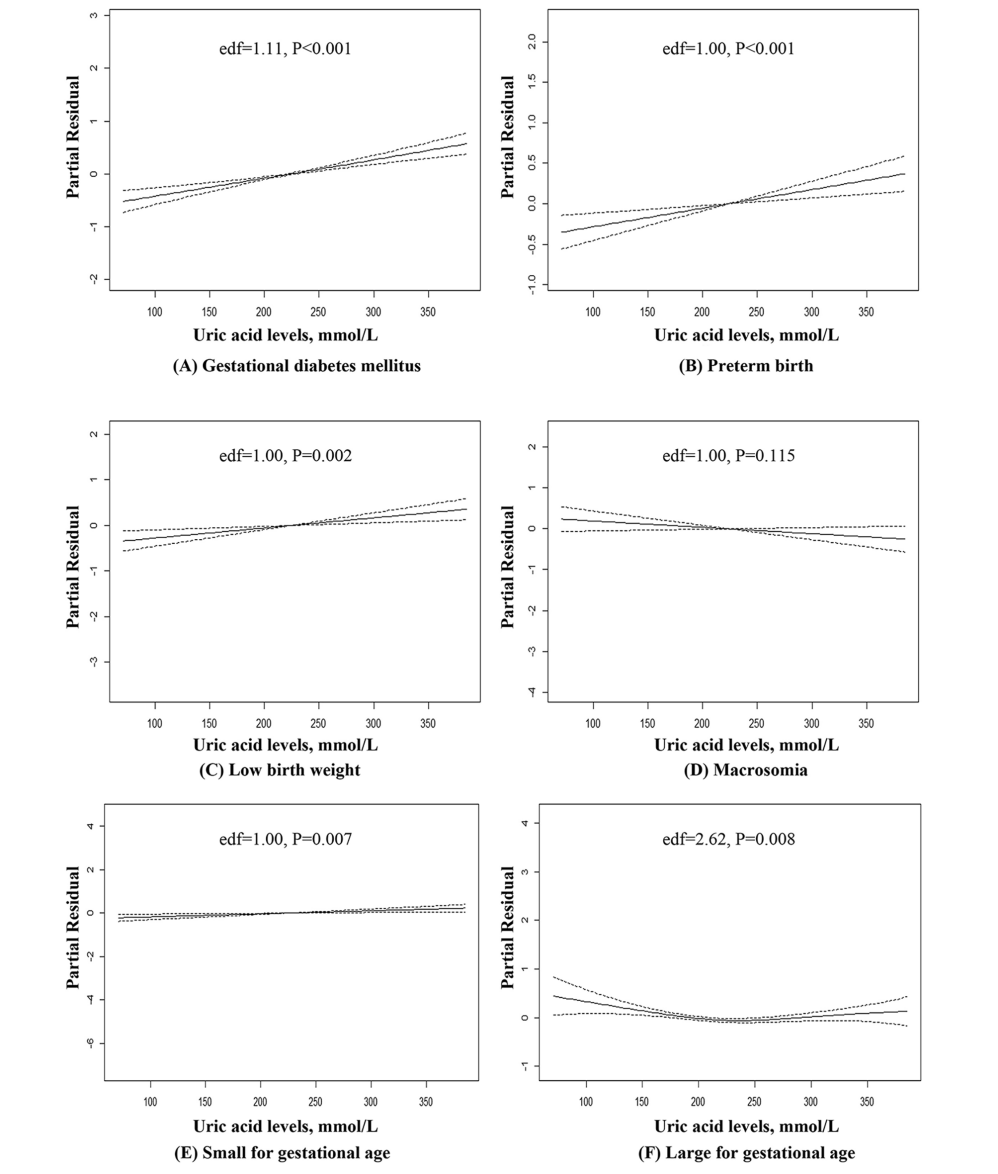
The relationships between early pregnant serum uric acid levels and outcomes of gestational diabetes mellitus (**A**), preterm birth (**B**), low birth weight (**C**), macrosomia (**D**), small for gestational age (**E**), and large for gestational age (**F**) based on the generalised additive regression models (n = 18,250). The covariates were maternal age, BMI, parity, and gestational weeks of uric acid measurement. Dotted lines represented the 95% confidence intervals. When the equivalent degrees of freedom (edf) value was close to 1.0, the relationship between uric acid and outcome tended to be linear. When the edf value was further from 1.0, the relationship between uric acid and outcome tended to be non-linear. A-p value of less than 0.05 indicates a statistical association between uric acid and outcome in the GAMs model

After adjustment for several potential covariates, higher UA levels in early pregnancy were associated with several adverse outcomes (Table 2). The ORs (95%CI) per one SD increase in UA levels were 1.205 (1.136, 1.277) for GDM, 1.137 (1.060, 1.221) for PB, 1.134 (1.051, 1.223) for LBW, and 1.077 (1.020, 1.137) for SGA, respectively. Similarly, hyperuricemia (vs. normal) was associated with a 39.4% (OR: 1.394, 95%CI: 1.211–1.606) higher risk of GDM, and a 38.5% (OR:1.385, 95%CI: 1.168–1.643) higher risk of PB, but not with LBW, macrosomia, SGA, and LGA. In the analysis of GAMs, when the equivalent degrees of freedom (edf) value was closer to 1.0, the relationship between uric acid and outcome tended to be linear. When the edf value was further from 1.0, the relationship between uric acid and outcome tended to be non-linear. A p-value of less than 0.05 indicates a statistical association between uric acid and outcome in the GAMs model. Serum UA levels showed linear relationships with GDM (edf = 1.11, P < 0.001), PB (edf = 1.00, P < 0.001), LBW (edf = 1.00, P = 0.002), and SGA (edf = 1.00, P = 0.007) based on GAMs (Fig. 2). The association between UA and LGA tended to be non-linear (edf = 2.62, P = 0.008). No significant associations were found between UA and macrosomia (edf = 1.00, P = 0.115).

**Table 2:** Associations between early pregnancy serum uric acid levels and gestational diabetes & adverse infant birth outcomes (N = 18,250)

	Model 1				Model 2		
	*OR*	*95%CI*	*P*		*OR*	*95%CI*	*P*
Hyperuricemia versus normal (reference)							
Gestational diabetes mellitus	1.386	(1.208, 1.589)	**< 0.001**		1.394	(1.211, 1.606)	**< 0.001**
Caesarean section	1.133	(1.046, 1.227)	**0.002**		1.057	(0.972, 1.148)	0.194
Premature birth	1.284	(1.084, 1.520)	**0.004**		1.385	(1.168, 1.643)	**< 0.001**
Low birth weight	1.060	(0.877, 1.281)	0.546		1.193	(0.985, 1.444)	0.070
Macrosomia	1.047	(0.812, 1.350)	0.722		0.837	(0.646, 1.084)	0.178
Small for gestational age	1.024	(0.893, 1.174)	0.734		1.124	(0.978, 1.291)	0.100
Large for gestational age	1.191	(1.06, 1.333)	**0.002**		1.098	(0.980, 1.231)	0.108
Per one SD increase of uric acid levels							
Gestational diabetes mellitus	1.159	(1.095, 1.227)	**< 0.001**		1.205	(1.136, 1.277)	**< 0.001**
Caesarean section	1.048	(1.016, 1.081)	**0.003**		1.018	(0.985, 1.052)	0.285
Premature birth	1.081	(1.008, 1.159)	**0.028**		1.137	(1.060, 1.221)	**< 0.001**
Low birth weight	1.050	(0.975, 1.131)	0.200		1.134	(1.051, 1.223)	**0.001**
Macrosomia	1.031	(0.933, 1.140)	0.544		0.918	(0828, 1.017)	0.101
Small for gestational age	1.029	(0.976, 1.086)	0.289		1.077	(1.020, 1.137)	**0.008**
Large for gestational age	1.015	(0.970, 1.062)	0.523		0.976	(0.932, 1.023)	0.317

Model 1: without adjustment

Model 2: adjusted for maternal age, BMI, parity, and gestational weeks of uric acid measurement

No significant interactions were observed between higher serum UA levels (both hyperuricemia or per one SD increase in UA concentration) and the gestational age of UA measurement on the risk of adverse outcome, as shown in Table 3. As shown in Table 4, significant interactions were observed between higher serum UA levels (per one SD increase in UA concentration) and the BMI groups (< 24, ≥ 24 and < 28, ≥28 kg/m^2^) on the risk of PB (P interaction = 0.001), and LBW (P interaction = 0.008). Significant interactions were observed between hyperuricemia and the BMI groups (< 24, ≥ 24 and < 28, ≥28 kg/m^2^) on the risk of SGA (P interaction = 0.028). No significant interactions were found between higher serum UA levels and age (< 35 and ≥ 35 years) or parity (0 and ≥ 1) on the risk of all outcomes, and the p-values of the interaction ranged from 0.097 to 0.826 and from 0.133 to 0.986, respectively (data not shown).

**Table 3:** Associations between early pregnancy serum uric acid levels and gestational diabetes & adverse infant birth outcomes stratified by gestational weeks of measurement

	< 17 weeks (N = 6,613)				≥ 17 weeks (N = 11,637)			Crude P-interaction	P-interaction
	*OR*	*95%CI*	*P*		*OR*	*95%CI*	*P*		
Hyperuricemia versus normal (reference)									
Gestational diabetes mellitus	1.365	(1.100, 1.695)	**0.005**		1.417	(1.175, 1.707)	**< 0.001**	0.756	0.834
Premature birth	1.338	(1.012, 1.768)	**0.041**		1.414	(1.140, 1.754)	**0.002**	0.709	0.777
Low birth weight	1.122	(0.821, 1.534)	0.471		1.238	(0.973, 1.577)	0.083	0.403	0.630
Macrosomia	0.981	(0.655, 1.469)	0.925		0.760	(0.542, 1.066)	0.112	0.758	0.472
Small for gestational age	1.151	(0.911, 1.455)	0.239		1.112	(0.936, 1.321)	0.226	0.919	0.970
Large for gestational age	1.025	(0.849, 1.239)	0.795		1.143	(0.990, 1.319)	0.068	0.486	0.395
Per one SD increase in uric acid concentration									
Gestational diabetes mellitus	1.235	(1.129, 1.351)	**< 0.001**		1.183	(1.094, 1.278)	**< 0.001**	0.676	0.419
Premature birth	1.166	(1.039, 1.308)	**0.009**		1.120	(1.024, 1.225)	**0.013**	0.677	0.550
Low birth weight	1.197	(1.060, 1.352)	**0.004**		1.095	(0.995, 1.206)	0.063	0.364	0.241
Macrosomia	0.982	(0.830, 1.162)	0.835		0.884	(0.777, 1.007)	0.063	0.398	0.512
Small for gestational age	1.098	(0.999, 1.206)	0.052		1.068	(0.999, 1.142)	0.053	0.828	0.786
Large for gestational age	0.928	(0.858, 1.003)	0.060		1.004	(0.948, 1.065)	0.884	0.184	0.150

The mean (sd) gestational weeks were 14.7 (1.89), and 18.3 (0.83) for the < 17 weeks, and the ≥ 17 weeks groups, respectively

The associations were adjusted for maternal age, BMI, parity, and gestational weeks of uric acid measurement

Crude P-interaction: P-interaction calculated without adjustment of other covariates

P-interaction: P-interaction calculated with full adjustment of covariates

**Table 4:** Associations between early pregnancy serum uric acid levels and gestational diabetes & adverse infant birth outcomes stratified by BMI

	BMI < 24 kg/m^2^ (N = 4,727)				24 ≤ BMI < 28 kg/m^2^ (N = 9,302)				BMI ≥ 28 kg/m^2^ (N = 4,221)			Crude P-interaction	P-interaction
	*OR*	*95%CI*	*P*		*OR*	*95%CI*	*P*		*OR*	*95%CI*	*P*		
Hyperuricemia versus normal (reference)													
Gestational diabetes mellitus	1.149	(0.823, 1.604)	0.415		1.517	(1.238, 1.859)	**< 0.001**		1.351	(1.057, 1.725)	**0.016**	0.294	0.467
Caesarean section	1.039	(0.866, 1.246)	0.681		1.057	(0.938, 1.190)	0.365		1.057	(0.907, 1.232)	0.475	0.224	0.700
Premature birth	1.269	(0.928, 1.736)	0.136		1.221	(0.939, 1.589)	0.136		1.806	(1.296, 2.515)	**< 0.001**	0.081	0.064
Low birth weight	0.931	(0.674, 1.285)	0.663		1.419	(1.069, 1.885)	**0.016**		1.196	(0.769, 1.859)	0.426	0.115	0.076
Macrosomia	1.143	(0.440, 2.967)	0.784		0.847	(0.553, 1.297)	0.445		0.822	(0.583, 1.159)	0.264	0.738	0.523
Small for gestational age	0.930	(0.730, 1.184)	0.556		1.177	(0.962, 1.440)	0.113		1.383	(1.008, 1.896)	**0.045**	0.074	**0.028**
Large for gestational age	1.016	(0.766, 1.347)	0.911		1.070	(0.899, 1.272)	0.446		1.148	(0.957, 1.378)	0.138	0.169	0.355
Per one SD increase in uric acid concentration													
Gestational diabetes mellitus	1.177	(1.041, 1.332)	**0.009**		1.213	(1.114, 1.320)	**< 0.001**		1.199	(1.075, 1.337)	**0.001**	0.619	0.679
Caesarean section	1.022	(0.955, 1.093)	0.530		1.001	(0.956, 1.048)	0.972		1.044	(0.978, 1.115)	0.196	0.117	0.342
Premature birth	1.002	(0.885, 1.133)	0.978		1.146	(1.032, 1.273)	**0.011**		1.330	(1.140, 1.552)	**< 0.001**	**0.002**	**0.001**
Low birth weight	1.027	(0.915, 1.154)	0.646		1.191	(1.059,1.339 )	**0.003**		1.247	(1.031, 1.509)	**0.023**	**0.019**	**0.008**
Macrosomia	0.934	(0.644, 1.357)	0.721		0.938	(0.799, 1.100)	0.430		0.909	(0.787, 1.049)	0.193	0.276	0.609
Small for gestational age	1.022	(0.936, 1.116)	0.629		1.107	(1.022, 1.199)	**0.013**		1.126	(0.977, 1.298)	0.100	0.247	0.110
Large for gestational age	0.954	(0.859, 1.060)	0.384		0.960	(0.896, 1.028)	0.245		1.001	(0.924, 1.085)	0.980	0.139	0.322

The associations were adjusted for maternal age, BMI, parity, and gestational weeks of uric acid measurement

Crude P-interaction: P-interaction calculated without adjustment of other covariates

P-interaction: P-interaction calculated with full adjustment of covariates

## Discussion

In this study based on retrospective data from an 18,250 mother-infants cohort in southern China, we observed that higher serum UA (both as a categorical and continuous variable) in early pregnancy was associated with higher risk of GDM, PB, LBW, and SGA. The dose-response models tended to be linear for most outcomes. Results tended to be more pronounced in subjects with higher BMI for the outcomes of PB, LBW, and SGA, with significant interactions being found for these associations.

The prevalence of hyperuricemia in our study was 15.9% (subjects), which was relatively higher than that of nonpregnant Chinese adults in a study of 5,939 subjects (14.1%) [30] and a study of 11,601 subjects (11.15%) in Shanghai City [31]. Serum UA concentrations increased during pregnancy and are affected by gestational age, which may partly explain why the prevalence of hyperuricemia was higher than that in nonpregnant subjects, although we tried to adjust hyperuricemia for gestational age. However, the prevalence of hyperuricemia in our study was relatively lower when compared with another study of 404 normotensive singleton women (103 subjects, 25.4%) [15]. The prevalence of hyperuricemia was barely mentioned in the previous study, moreover, hyperuricemia was common defined as ≥ 1 SD values at corresponding age, was sample-based, and UA was measured at different trimesters. These increased the difficulty in comparing the prevalence of hyperuricemia among different populations, and these issues should be further considered in the future.

The positive associations found in our study were consistent with several previous studies. Higher UA levels (or hyperuricemia) in the first 20 weeks of pregnancy were associated with a 2.34- and 1.11- fold increased risk of GDM in 1000 Chinese [11] and 5507 Israeli women [12], respectively. Similar results have been observed in a retrospective cohort study of 23,843 Chinese with UA measured before 24 weeks of gestation [32] and other studies [10, 13]. However, several studies also reported marginal or null associations between UA and GDM [18, 20, 22]. Adverse associations with PB, SGA, or BW were also found in several studies with small sample size of less than 500 [14–16], which were consistent with ours. In a retrospective study of 11,580 Chinese, higher UA in late pregnancy (the corresponding gestational weeks at delivery) was associated with a higher risk of LBW, SGA, but also associated with a lower risk of PB [33]. Marginal or null significant results were found in a cross-sectional study of 885 Germans [19] and in a prospective cohort of 1541 Americans [21]. In another prospective cohort of 1291 Americans, both too high and too low UA levels were detrimental to fetal growth [17]. The UA measured in different trimesters or periods of pregnancy may partly explain the heterogeneity of these results, as UA levels tend to be influenced by the course of pregnancy [27]. In our study, linear rather than U-shaped relationships were observed for UA and most gestational or birth outcomes. Highlighting the associations with adverse outcomes of UA in early pregnancy instead of late pregnancy, may provide a better opportunity for pregnant women to improve their UA status and possibly partially prevent the occurrence of adverse outcomes. In addition, the longer time between UA measurement (early versus late pregnancy) and outcomes increases the ability to avoid the possibility of causal inversion. Our study included a large number of subjects, which may reduce the possibility of false-negative results that may have occurred in previous studies with much smaller sample sizes. Our results, along with several, but not all, studies, underscore the importance of UA in early pregnancy as an indicator of GDM and adverse birth outcomes, even in normotensive subjects.

Several mechanisms may contribute to the adverse associations of UA. Higher UA might affect GDM by increasing the risk of insulin resistance during pregnancy [34], through several potential pathways, including endothelial dysfunction [35], decreased production of nitric oxide [36, 37], and induction of metabolic inflammation [38]. Apart from the mechanisms mentioned above, elevated UA might inhibit amino acid uptake in the placenta [39], and attenuate trophoblast invasion and integration into endothelial cell monolayers [40], leading to poor placental development. Most of these mechanisms have also been implicated in the development of adverse gestational cardiovascular complications. Because adverse associations were observed in pregnant women without the occurrence of cardiovascular complications, other potential mechanisms may exist, and need to be well characterized by further studies.

More pronounced adverse associations were found in subjects with higher BMI for outcomes of PB, LBW, and SGA. Indeed, obesity is one of the most important pathways for adverse outcomes [41]. Moreover, obesity also leads to higher UA levels [42, 43], although we tried to attenuate its influence by adjusting for BMI. Our results suggest that clinicians should make more precise considerations for subjects with both high UA levels and obesity in early pregnancy.

Our study has several strengths. The study was based on a large sample of 18,250 subjects, which provided more precise estimates of the associations. Serum UA data were measured and collected in early pregnancy, which established the temporal sequence of the observed associations. We examined the functional forms of the dose-response associations, and potential interactions from multiple variables, including age, BMI, UA measurement times, and parity. Our results provided further evidence for obstetric clinicians to identify higher UA levels and potential high-risk patients.

There are also several limitations to the study that deserve careful consideration. First, serum levels of UA were measured and recorded only once during the pregnancy in subjects without gestational cardiovascular complications, so we were unable to analyze the dynamic course of UA and its influence on outcomes during the early pregnancy. Second, the study was based on data from an obstetric center instead of a community, which might have weakened the representativeness of the sample, but this could partly be counteracted because the obstetric center was the largest one in the areas and received patients from different regions of the city. In addition, it would be of very difficult to collect medical record information from the community for a retrospective study. Third, although we adjusted for several potential covariates, there may still be residual confounding.

## Conclusions

In conclusion, this retrospective cohort study found that higher UA levels in early pregnancy were associated with higher risk of GDM, PB, LBW, and SGA in normotensive Chinese women.

### Electronic supplementary material

Below is the link to the electronic supplementary material.

**Supplemental Table 1**. Distribution of serum uric acid concentrations (mmol/L) of different gestational age.

## Data Availability

The data that support the findings of this study are available from the Affiliated Foshan Maternity & Child Healthcare Hospital, Southern Medical University, but restrictions apply to the availability of these data, which were used under license for the current study, and so are not publicly available. Data are however available from the corresponding author upon reasonable request and with permission of Affiliated Foshan Maternity & Child Healthcare Hospital, Southern Medical University.

## List of abbreviations

UA: Uric acid
GDM: Gestational diabetes mellitus
PB: Preterm birth
LBW: Low birth weight
SGA: Small for gestational age
LGA: Large for gestational age
BMI: Body mass index
SD: Standard deviation
OR: Odds ratio
CI: Confidence interval
GAM: Generalised additive regression models

## References

[1] Jun JE, Lee YB, Lee SE, Ahn JY, Kim G, Jin SM, Hur KY, Lee MK, Kang MR, Kim JH (2018). Elevated serum uric acid predicts the development of moderate coronary artery calcification Independent of conventional cardiovascular risk factors. Atherosclerosis.

[2] Strasak AM, Kelleher CC, Brant LJ, Rapp K, Ruttmann E, Concin H, Diem G, Pfeiffer KP, Ulmer H (2008). Serum uric acid is an Independent predictor for all major forms of cardiovascular death in 28,613 elderly women: a prospective 21-year follow-up study. Int J Cardiol.

[3] Jia Z, Zhang X, Kang S, Wu Y (2013). Serum uric acid levels and incidence of impaired fasting glucose and type 2 Diabetes Mellitus: a meta-analysis of cohort studies. Diabetes Res Clin Pract.

[4] Yuan H, Yu C, Li X, Sun L, Zhu X, Zhao C, Zhang Z, Yang Z (2015). Serum uric acid levels and risk of metabolic syndrome: a dose-response Meta-analysis of prospective studies. J Clin Endocrinol Metab.

[5] Liu CQ, He CM, Chen N, Wang D, Shi X, Liu Y, Zeng X, Yan B, Liu S, Yang S, Li X, Li X, Li Z (2016). Serum uric acid is independently and linearly associated with risk of nonalcoholic fatty Liver Disease in obese Chinese adults. Sci Rep.

[6] Wolak T, Shoham-Vardi I, Sergienko R, Sheiner E (2015). High uric acid levels during pregnancy linked to increased risk for future atherosclerotic-related hospitalization. J Clin Hypertens (Greenwich).

[7] Schmella MJ, Clifton RG, Althouse AD, Roberts JM (2015). Uric acid determination in gestational Hypertension. Is it as effective a delineator of risk as Proteinuria in High-Risk women?. Reprod Sci.

[8] Matias ML, Romao M, Weel IC, Ribeiro VR, Nunes PR, Borges VT, Araujo JP, Peracoli JC, de Oliveira L, Peracoli MT (2015). Endogenous and Uric Acid-Induced activation of NLRP3 inflammasome in pregnant women with Preeclampsia. PLoS ONE.

[9] Hawkins TL, Roberts JM, Mangos GJ, Davis GK, Roberts LM, Brown MA (2012). Plasma uric acid remains a marker of poor outcome in hypertensive pregnancy: a retrospective cohort study. BJOG.

[10] Zhao H, Li H, Chung ACK, Xiang L, Li X, Zheng Y, Luan H, Zhu L, Liu W, Peng Y, Zhao Y, Xu S, Li Y, Cai Z (2019). Large-scale Longitudinal Metabolomics Study reveals different trimester-specific alterations of metabolites in Relation to Gestational Diabetes Mellitus. J Proteome Res.

[11] Zhou J, Zhao X, Wang Z, Hu Y (2012). Combination of lipids and uric acid in mid-second trimester can be used to predict adverse pregnancy outcomes. J Matern Fetal Neonatal Med.

[12] Wolak T, Sergienko R, Wiznitzer A, Paran E, Sheiner E (2012). High uric acid level during the first 20 weeks of pregnancy is associated with higher risk for gestational Diabetes Mellitus and mild preeclampsia. Hypertens Pregnancy.

[13] Tisi DK, Burns DH, Luskey GW, Koski KG (2011). Fetal exposure to altered amniotic fluid glucose, insulin, and insulin-like growth factor-binding protein 1 occurs before screening for gestational Diabetes Mellitus. Diabetes Care.

[14] Le TM, Nguyen LH, Phan NL, Le DD, Nguyen HVQ, Truong VQ, Cao TN (2019). Maternal serum uric acid concentration and pregnancy outcomes in women with pre-eclampsia/eclampsia. Int J Gynaecol Obstet.

[15] Amini E, Sheikh M, Hantoushzadeh S, Shariat M, Abdollahi A, Kashanian M (2014). Maternal hyperuricemia in normotensive singleton pregnancy, a prenatal finding with continuous perinatal and postnatal effects, a prospective cohort study. BMC Pregnancy Childbirth.

[16] Gao T, Zablith NR, Burns DH, Skinner CD, Koski KG (2008). Second trimester amniotic fluid transferrin and uric acid predict infant birth outcomes. Prenat Diagn.

[17] Zhou G, Holzman C, Luo Z, Margerison C. Maternal serum uric acid levels in pregnancy and fetal growth. J Matern Fetal Neonatal Med 2018 Jul:1–9. 10.1080/14767058.2018.1484093. 10.1080/14767058.2018.1484093 29961396

[18] Correa PJ, Venegas P, Palmeiro Y, Albers D, Rice G, Roa J, Cortez J, Monckeberg M, Schepeler M, Osorio E, Illanes SE (2019). First trimester prediction of gestational Diabetes Mellitus using plasma biomarkers: a case-control study. J Perinat Med.

[19] Rothenbacher D, Braig S, Logan CA (2018). Association of maternal uric acid and cystatin C serum concentrations with maternal and neonatal cardiovascular risk markers and neonatal body composition: the Ulm SPATZ Health Study. PLoS ONE.

[20] Maged AM, Moety GA, Mostafa WA, Hamed DA (2014). Comparative study between different biomarkers for early prediction of gestational Diabetes Mellitus. J Matern Fetal Neonatal Med.

[21] Laughon SK, Catov J, Powers RW, Roberts JM, Gandley RE (2011). First trimester uric acid and adverse pregnancy outcomes. Am J Hypertens.

[22] Gungor ES, Danisman N, Mollamahmutoglu L (2006). Relationship between serum uric acid, creatinine, albumin and gestational Diabetes Mellitus. Clin Chem Lab Med.

[23] Dong XW, Tian HY, He J, Wang C, Qiu R, Chen YM (2016). Elevated serum uric acid is Associated with Greater Bone Mineral density and skeletal muscle Mass in Middle-aged and older adults. PLoS ONE.

[24] Huang R, Tian S, Han J, Lin H, Guo D, Wang J, An K, Wang S (2019). U-Shaped Association between serum uric acid levels and cognitive functions in patients with type 2 Diabetes: a cross-sectional study. J Alzheimers Dis.

[25] Tseng WC, Chen YT, Ou SM, Shih CJ, Tarng DC (2018). U-Shaped Association between serum uric acid levels with Cardiovascular and all-cause mortality in the Elderly: the role of Malnourishment. J Am Heart Assoc.

[26] Cho SK, Chang Y, Kim I, Ryu S (2018). U-Shaped Association between serum uric acid level and risk of mortality: a Cohort Study. Arthritis Rheumatol.

[27] Lind T, Godfrey KA, Otun H, Philips PR (1984). Changes in serum uric acid concentrations during normal pregnancy. BJOG.

[28] National Health Commission of the People’s Republic of China. Growth standard for newborns by gestational age (WS/T 800–2022). 2022. http://www.nhc.gov.cn/wjw/fyjk/202208/d6dcc281e9b74db88dc972b34cbd3ec7.shtml. Accessed 20 Oct 2023.

[29] Revision Committee of Guidelines for Prevention and Control of Overweight and Obesity in Adults in China: Guidelines for Adult Overweight and Obesity Prevention and Control in China. (2021). Beijing: People’s Medical Publishing House 2021.

[30] Zeng C, Guo B, Wan Y, Guo Y, Chen G, Duoji Z, Qian W, Danzhen W, Meng Q, Chen L (2022). The role of lipid profile in the relationship between particulate matters and hyperuricemia: a prospective population study. Environ Res.

[31] Tao M, Ma X, Pi X, Shi Y, Tang L, Hu Y, Chen H, Zhou X, Du L, Chi Y (2021). Prevalence and related factors of hyperuricaemia in Shanghai adult women of different ages: a multicentre and cross-sectional study. BMJ Open.

[32] Yue C, Ying C, Li X (2023). Elevated serum uric acid is Associated with Gestational Diabetes Mellitus: an Observational Cohort Study. J Clin Endocrinol Metab.

[33] Yuan X, Han X, Jia C, Wang H, Yu B. Association of Maternal Serum Uric Acid and Cystatin C Levels in Late Pregnancy with Adverse Birth Outcomes: An Observational Cohort Study in China. Int J Womens Health 2022, 14:213–223. 10.2147/IJWH.S350847. eCollection 2022. 10.2147/IJWH.S350847 PMC8860627 35210868

[34] Laughon SK, Catov J, Roberts JM (2009). Uric acid concentrations are associated with insulin resistance and birthweight in normotensive pregnant women. Am J Obstet Gynecol.

[35] Feig DI, Kang DH, Johnson RJ (2008). Uric acid and cardiovascular risk. N Engl J Med.

[36] Roy D, Perreault M, Marette A (1998). Insulin stimulation of glucose uptake in skeletal muscles and adipose tissues in vivo is NO dependent. Am J Physiol.

[37] Bainbridge SA, Roberts JM. Uric acid as a pathogenic factor in preeclampsia. Placenta 2008;29 suppl A:S67-72. 10.1016/j.placenta.2007.11.001. 10.1016/j.placenta.2007.11.001 PMC3319018 18093648

[38] Kushiyama A, Nakatsu Y, Matsunaga Y, Yamamotoya T, Mori K, Ueda K, Inoue Y, Sakoda H, Fujishiro M, Ono H, Asano T (2016). Role of uric acid metabolism-related inflammation in the pathogenesis of metabolic Syndrome Components such as Atherosclerosis and Nonalcoholic Steatohepatitis. Mediators Inflamm.

[39] Bainbridge SA, von Versen-Hoynck F, Roberts JM (2009). Uric acid inhibits placental system a amino acid uptake. Placenta.

[40] Bainbridge SA, Roberts JM, von Versen-Hoynck F, Koch J, Edmunds L, Hubel CA (2009). Uric acid attenuates trophoblast invasion and integration into endothelial cell monolayers. Am J Physiol Cell Physiol.

[41] Han Q, Shao P, Leng J, Zhang C, Li W, Liu G, Zhang Y, Li Y, Li Z, Ren Y, Chan JCN, Yang X (2018). Interactions between general and central obesity in predicting gestational Diabetes Mellitus in Chinese pregnant women: a prospective population-based study in Tianjin. China J Diabetes.

[42] Tsushima Y, Nishizawa H, Tochino Y, Nakatsuji H, Sekimoto R, Nagao H, Shirakura T, Kato K, Imaizumi K, Takahashi H, Tamura M, Maeda N, Funahashi T, Shimomura I (2013). Uric acid secretion from adipose tissue and its increase in obesity. J Biol Chem.

[43] Ishiro M, Takaya R, Mori Y, Takitani K, Kono Y, Okasora K, Kasahara T, Tamai H (2013). Association of uric acid with obesity and endothelial dysfunction in children and early adolescents. Ann Nutr Metab.

