# Supplementary material for: Associations of early pregnancy serum uric acid levels with risk of gestational diabetes and birth outcomes: a retrospective cohort study
Source: BMC Endocr Disord. 2023 Nov 20;23:252. doi: 10.1186/s12902-023-01502-3 (PMC10658968; doi:10.1186/s12902-023-01502-3)
Supplement: Supplementary file 1 — Supplemental Table 1. Distribution of serum uric acid concentrations (mmol/L) of different gestational age. [file 12902_2023_1502_MOESM1_ESM.docx]

**Supplemental Table 1. Distribution of serum uric acid concentrations (mmol/L) of different gestational age.**

| Gestational weeks | N | Mean | SD | Mean +1 SD | Hyperuricemia,  N (%) |
| --- | --- | --- | --- | --- | --- |
| 6~12 | 569 | 0.2144 | 0.0487 | 0.2631 | 84 (14.8) |
| >12~13 | 1478 | 0.2154 | 0.0494 | 0.2648 | 242 (16.4) |
| >13~14 | 818 | 0.2129 | 0.0482 | 0.2611 | 128 (15.6) |
| >14~15 | 318 | 0.2209 | 0.0486 | 0.2695 | 60 (18.9) |
| >15~16 | 776 | 0.2210 | 0.0505 | 0.2715 | 127 (16.4) |
| >16~17 | 3215 | 0.2232 | 0.0486 | 0.2718 | 501 (15.6) |
| >17~18 | 4300 | 0.2260 | 0.0498 | 0.2758 | 691 (16.1) |
| >18~19 | 4272 | 0.2282 | 0.0506 | 0.2788 | 673 (15.8) |
| >19~20 | 2504 | 0.2281 | 0.0506 | 0.2787 | 390 (15.6) |

Hyperuricemia was defined as subjects with serum concentration of uric acid ≥ Mean +1 SD at the corresponding gestational age.
